# Analytical method for calculation of deviations from intended dosages during multi-infusion

**DOI:** 10.1186/s12938-016-0309-4

**Published:** 2017-01-17

**Authors:** Maurits K. Konings, Roland A. Snijder, Joris H. Radermacher, Annemoon M. Timmerman

**Affiliations:** Department of Medical Technology and Clinical Physics, University Medical Center Utrecht, Room AZU - C.01.230, P.O. Box 85500, 3508 GA Utrecht, The Netherlands

**Keywords:** Infusion, Catheter, Dosing error, Mathematical model, Safety, Poiseuille flow

## Abstract

**Background:**

In this paper, a new method is presented that combines mechanical compliance effects with Poiseuille flow and push-out effects (“dead volume”) in one single mathematical framework for calculating dosing errors in multi-infusion set-ups. In contrast to existing numerical methods, our method produces explicit expressions that illustrate the mathematical dependencies of the dosing errors on hardware parameters and pump flow rate settings.

**Methods:**

Our new approach uses the Z-transform to model the contents of the catheter, and after implementation in Mathematica (Wolfram), explicit expressions are produced automatically. Consistency of the resulting analytical expressions has been examined for limiting cases, and three types of in-vitro measurements have been performed to obtain a first experimental test of the validity of the theoretical results.

**Results:**

The relative contribution of various factors affecting the dosing errors, such as the Poiseuille flow profile, resistance and internal volume of the catheter, mechanical compliance of the syringes and the various pump flow rate settings, can now be discerned clearly in the structure of the expressions generated by our method. The in-vitro experiments showed a standard deviation between theory and experiment of 14% for the delay time in the catheter, and of 13% for the time duration of the dosing error bolus.

**Conclusions:**

Our method provides insight and predictability in a large range of possible situations involving many variables and dependencies, which is potentially very useful for e.g. the development of a fast, bed-side tool (“calculator”) that provides the clinician with a precise prediction of dosing errors and delay times interactively for many scenario’s. The interactive nature of such a device has now been made feasible by the fact that, using our method, explicit expressions are available for these situations, as opposed to conventional time-consuming numerical simulations.

## Background

If a patient needs intravenous administration of medication, it is important for the clinician to understand the basic pharmacokinetics of these medications. However, in recent years, ample evidence has been found [1–4], including a number of clinical cases and in vivo studies [5–9], indicating that physical effects related to the infusion hardware are equally important to understand [10, 11]. Especially in critical care, on the ICU and the OR, where multiple medications are typically delivered through one single lumen of a thin catheter, these physical effects may cause ambiguous and counter-intuitive discrepancies between the intended dose and the dose that has been actually delivered. This is particularly true if the dosing rate is adjusted ad hoc [12]; for example, fast-acting and critical inotropic drugs are often titrated, based on the mean arterial blood pressure (MABP).

There are three major factors that can produce significant deviations from the intended medication dosing rate scheme: (i) the length of the catheter causes a delay in the administration of the medication into the patient, and therefore, a mixture, corresponding to previous medication dosing rates, may still be present inside the catheter, i.e. the contents inside the catheter constitutes a “memory” in which the effects of previous medication dosing rates may be stored. This has been called “dead volume effect” in the literature [7, 13]. (ii) The syringes that are used in clinical practice are far from ideal, because these syringes have a significant mechanical compliance primarily due to the compressibility of the rubber plunger inside the syringe [14]. Therefore, whenever the flow rate setting of one of the infusion pumps in a multi-infusion set-up is altered, pressure changes within the entire system cause a change in the deformation of compressible and expandable parts (i.e., the other syringes) within the system, and hence may cause a deviation from the intended flow rates. (iii) The low velocities of the fluids in catheters ensure that the flow inside these lines will be laminar (low Reynolds number), and hence exhibit a Poiseuille flow profile, in which the fluid particles near the central longitudinal axis of the catheter travel faster than fluid particles near the wall of the catheter, thus giving rise to a “mixing effect” of its own.

Several infusion simulation studies have shown the importance of these problems by demonstrating the influence of the physical effects of dead volume, compliance and the Poiseuille effect on drug delivery in isolation: There are a number of studies that describe the “dead volume effect” in isolation from the syringe compressibility (“compliance”) effect [7]. In simple cases, calculation of the “dead volume effect” is straightforward: If the actual volumetric flow rate $u_{cath}$ inside a single lumen catheter is assumed to be constant, and the internal volume $V_{cath}$ of the catheter (i.e., the internal volume as measured starting from the mixing point, at which the medications from all syringes come together, up to the distal catheter tip inside the vasculature of a patient) is known, then calculation of the “delay time” $t_{delay}$, i.e. the time needed for a droplet of medication to travel through the dead volume before it reaches the blood stream of the patient, is simply $t_{delay} = V_{cath}/u_{cath}$.

In clinical practice, however, the situation is often more complex: the flow rate may vary during the delay time due to changes in the pump flow rate settings, and the actual flow rate also typically differs from the pump flow rate setting value temporarily, due to the effects of mechanical compliance as mentioned above. These compliance effects have been studied in isolation as well [2, 3, 15]. Its basic mechanism is a “capacitor” effect, which has been quantified using the electric analog of a system containing capacitors, and the calculations of the dosing errors have been performed using the Laplace transform.

The third major factor, the mixing effect due to the Poiseuille profile [16], can be described as a convolution, as will be explained in more detail in “Appendix”. It causes a spreading out of the dosing error in time, in which the first arrival of the dosing error occurs sooner then it would have without Poiseuille mixing effect.

As a result, due to the combination of the effects (i), (ii), and (iii) mentioned above, non-trivial deviations from the intended medication dosages scheme may occur, as will be explained below.

In an earlier paper, we have shown that the “dead volume effect” on one hand, and syringe compliance (“capacitor”) effect on the other hand, produce opposite deviations from the pump flow rate settings in the actual drug output concentrations, making the net result hard to predict and often counterintuitive [4].

In this paper, however, we focus on the mathematical method of calculating dosing errors in multi-infusion setups in complex situations. The aim of the new method described in this paper is to obtain analytical expressions for the deviations from the intended dosages during multi-infusion that result from the combination of the effects (i) and (ii) described above. These analytical expressions contain dependencies on parameters that represent the physical variables in a multi-infusion set-up, such as: intended flow rates of the various pumps, compliances of the syringes, resistances of the various tubes, height differences within the multi-infusion set-up, etc.

As opposed to conventional numerical simulations, our objective therefore is to obtain explicit analytical expressions for these deviations, in which physical characteristics of the multi-infusion set-up are represented explicitly as variables.

We see the need for a tool for clinicians and medical physicist that provides understanding of the role that various physical parameters (i.e., characteristics of the multi-infusion hardware) play in the emergence of a dosing error. In order to make these roles explicit, we will use our new mathematical approach, presented in the “Method: analytical model, and in-vitro set-up” section of this paper, to explicate these roles in the form of direct mathematical relationships between physical hardware parameters and dosing errors.

For our mathematical model we used the strong symbolic calculation capabilities of the Mathematica package (Mathematica 10, Wolfram^®^ Inc., USA) to process Laplace-transforms and Z-transforms analytically. This will be explained in “Method: analytical model, and in-vitro set-up” section. Furthermore, we have performed in-vitro measurements in order to verify our mathematical results experimentally.

## Method: analytical model, and in-vitro set-up 

**Fig. 1:**
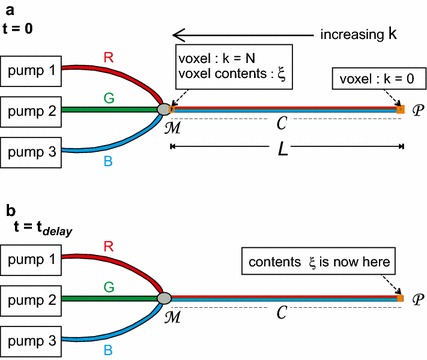
Example of a general multi-infusion set-up. In this example, the number of pumps is three. The method, however, allows for any number of pumps. **a** The catheter $\mathcal {C}$ contains a mixture of the fluids “R”, “G”, and “B”, in which “R”, “G”, and “B” are the contents of syringe 1, syringe 2, and syringe 3, respectively. We consider this mixture to be completely homogeneous, despite the “stratified” rendering of the *three colours* in catheter $\mathcal {C}$ in this figure. **b** Situation at $t=t_{delay}$; since $t_{delay}$ is by definition the time needed to travel through the catheter $\mathcal {C}$, the contents $\xi$ that was inside the voxel $k=N$ (near the mixing point $\mathcal {M}$) at time $t = 0$ in **a**, has now reached the very tip $\mathcal {P}$ of the catheter at time $t=t_{delay}$, and is entering the blood stream of the patient

To start with, we analyse the total chain of causality, from a change in the pump flow rate setting value, up to the moment that a dosing error enters the bloodstream of the patient. As has been mentioned in the introduction, it is essential to recognize that the lumen of the catheter (i.e. the internal volume $\mathcal {C}$ inside the catheter, starting from the mixing point $\mathcal {M}$, at which the medications from all syringes come together, up to the distal tip $\mathcal {P}$ of the infusion line inside the vasculature of a patient) constitutes a *memory* in which the effects of previous changes in pump flow rate settings are stored. In the explanation of our method, we focus on a single-lumen catheter. This however can be easily extended to a multi-lumen catheter. In order to represent this memory in the mathematical model, the internal volume of the catheter $\mathcal {C}$ between the points $\mathcal {M}$ and $\mathcal {P}$ is divided into tiny voxels $A_k$, in which the index *k* runs from $k=0$ at point $\mathcal {P}$ to $k=N$ at point $\mathcal {M}$ (see Fig. 1), in which *N* is a very large number. Let *L* denote the length of the catheter. Hence, the length of a single voxel equals $\gamma$, with $\gamma = L/N$. In our mathematical model, we use a general set-up containing any number of infusion pumps. For simplicity, however, we start with three pumps, in which each of these pumps contains a solution of a different medication, in which we used colours (“Red”, “Green”, and “Blue”, or R, G, and B, respectively) to denote the three different solutions. It is important to note that the R, G, and B denote the three different solutions as stored in their respective syringes, not the medications themselves. Furthermore, in this paper, the dosing errors will be expressed as volumes of the R, G, and B solutions, instead of dosages of the medications themselves. In Fig. 1a, the situation directly after $t=0$ is depicted, i.e. the point in time at which a change in pump flow rate setting value of one of the pumps takes place. As a result, the first small voxel at k=N near the mixing point $\mathcal {M}$ is now being filled with a droplet $\xi$ featuring a new mixing ratio between the solutions R, G, and B, resulting from the new pump flow rate setting values at $t=0$. The rest of the voxels inside the line $\mathcal {C}$, however, contain a fluid mixture that still has the old mixing ratio corresponding to the steady-state situation before the change in pump flow rate setting value. In Fig. 1b, the situation at $t=t_{delay}$ is depicted, i.e. the point in time at which this specific droplet $\xi$, that has been situated inside voxel $A_{k=N}$ at $t=0$, has now reached the distal tip of the catheter at point $\mathcal {P}$ at $t=t_{delay}$. Hence, at $t=t_{delay}$, the entire line $\mathcal {C}$ has now been filled with the new mixing ratio. The contents of voxel $A_k$, for any value of *k*, is described by the 3D-vector $\bar{a}_k$, in which

1 $$\begin{aligned} \bar{a}_k = \left( \begin{array}{cc} a_k^{(R)} \\ a_k^{(G)} \\ a_k^{(B)} \end{array} \right) \end{aligned}$$

in which

2 $$\begin{aligned} \forall k : \; \left( a_k^{(R)} + a_k^{(G)} + a_k^{(B)} = 1 \wedge 0 \le a_k^{(R)} \le 1 \wedge 0 \le a_k^{(G)} \le 1 \wedge 0 \le a_k^{(B)} \le 1 \right) \end{aligned}$$

The ratio $a_{k}^{(R)}$ represents the volume fraction of fluid (R) inside voxel *k*, i.e. the volume of solution (R) inside voxel *k* divided by the total volume inside voxel *k*. The voxel for which $k=0$ is the very last voxel at point $\mathcal {P}$ inside the catheter, i.e. the distal tip of the catheter. This specific voxel at $k=0$ releases its contents $\bar{a}_{k=0}$ directly into the blood stream of the patient.

Now consider the situation in which the flow rate setting of the “Green” pump is changed suddenly at $t=0$, whereas the flow rate settings of the “Red” and “Blue” pumps are never altered. If the “old” total flow rate inside the catheter $\mathcal {C}$ before $t=0$ has been constant and equal to $u_{old}$ for all $t<0$, and the “new” total flow rate is also constant and equals $u_{new}$ for all $t>0$, then the partial flow rate $u^{(R)}_{patient}$ of the “Red” fluid leaving the catheter at point $\mathcal {P}$ (and thus entering the patient) at $t=0$ equals:

3 $$\begin{aligned} u^{(R)}_{patient}(t) = u_{new} \, \cdot \, a_{(k=0)}^{(R)}(t) = u_{new} \, \cdot \, \left( \text{fraction } \text{ of } \text{(R) } \text{in } \text{the } \text{old } \text{mixing } \text{ratio} \right) \; \; \text{ for } t > 0 \text{ and } t < t_{delay} \end{aligned}$$

in which the “old” mixing ratio refers to the mixing ratio produced by the pump rate settings of $t<0$.

**Fig. 2:**
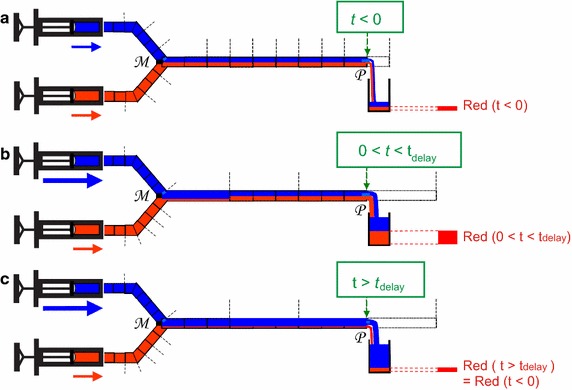
Schematic, illustrating the “push-out” effect, i.e.: the temporary increase in the outflow of the “red” fluid into the patient due to an increase in speed of the “blue” pump, as is explained below. **a** Two infusion pumps, filled with a “blue” and a “red” solution, respectively, produce the same flow rate, which is constant in time. The two flows are merged at the “mixing point” M, and subsequently travel towards point P, which represents the distal tip of the catheter where the fluid enters the blood stream of the patient. Since the flow rates of both pumps are equal, the catheter from point M to point P contains equal amounts of red and blue fluid, in the form of a mixture. This mixture is travelling with a flow rate that is twice the flow rate of each pump. A constant amount *r* of red fluid is entering the patient during each unit time interval (illustrated by a canister at point P for each unit time interval). **b** The same set-up, but now, at $\hbox {t} = 0$, the flow rate of the “blue” pump is suddenly increased by a factor 5. As a result, after $t=0$, the amount of red fluid entering the patient at point P now temporarily equals 3*r* per unit time. Therefore, in **b**, the “memory effect” is visible: the distal part of the catheter (near P) still contains the “old” mixing ratio corresponding to the situation before $t=0$. This “old” mixture is now being pushed out at the new speed; hence we dubbed this temporary increase in output of red fluid the “push-out” effect. In the literature, this effect is sometimes referred to as “dead volume” effect, or “catheter memory effect”. **c** After $\hbox {t} = \hbox {t}_{delay}$, the amount of red fluid entering the blood stream per unit time equals *r* again, which equals the intended dose, just like the situation before $t=0$ in **a**

As is explained in Fig. 2, the presence of the “old” mixing ratio in Eq. (3) can give rise to effects that may be unexpected to physicians, in which a temporary increase in the partial flow rate of (R) entering the blood stream occurs, although the setting value of the flow rate of the pump of (R) has never been changed. We will refer to this unwanted temporary increase in the $$u^{(R)}_{patient}$$ as the “push-out” effect, which has sometimes been called “dead volume” effect in the literature [7]. In Fig. 2a, the situation before $$t=0$$ is depicted. In Fig. 2b, a temporary, unplanned, and undesirable increase in $$ u^{(R)}_{patient}$$ occurs (see figure legend for explanation). After $$t=t_{delay}$$, the value of $$ u^{(R)}_{patient}$$ returns to its original value (Fig. 2c).

Furthermore, due to the mechanical compliance of the syringes, however (as mentioned at point (ii) in the introduction), the actual flow rate $u_{cath}(t)$ in the catheter does not follow the changes in pump flow rate setting values immediately. As a result, the actual flow rate $u_{cath}(t)$ is not exactly equal to the sum of the pump flow rate setting values $u_{pump}^{(R)}(t)$, $u_{pump}^{(G)}(t)$, and $u_{pump}^{(B)}(t)$, due to the transient mechanical compliance effects that cause extra fluid to be stored in, or released from, the syringes directly after changes in pump flow rate setting values:

4 $$\begin{aligned} u_{pump}^{(R)}(t) \, + \, u_{pump}^{(G)}(t) \, + \, u_{pump}^{(B)}(t) \; \ne \; u_{cath}(t) \end{aligned}$$

Therefore, the general expression from which the “delay time” $t_{delay}$ needs to be established is:

5 $$\begin{aligned} V_{cath} = \int _0^{t_{delay}} u_{cath}(\tau ) d\tau \end{aligned}$$

in which $u_{cath}$ depends on the compliance characteristics of the syringes as well.

### New method for incorporating the memory effect of the catheter into an analytical model

**Fig. 3:**
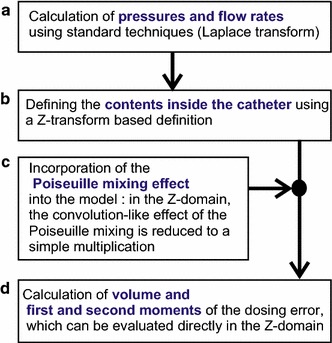
Outline of the analytical method as presented in this paper. **a** See “Starting point: Laplace transform and Kirchho’s laws” section. **b** See “Contents of the catheter without Poiseuille mixing effect” section. **c** See “Poiseuille mixing effect” section. **d** See “Calculation of moments of the dosing error distribution after T_*delay*_” section

In this paper, we will follow the following strategy to calculate dosing errors analytically (see Fig. 3):

First, standard techniques [17] (involving the Laplace Transform) are used to calculate pressures and total flow rates (see Fig. 3a), in which the differences between the “Red”, “Blue” and “Green” solutions are disregarded; i.e. in this “color-blind” calculation, only the total flow rate inside the catheter $u_{cath}(t)$ is calculated. The mechanical compliance of the catheter is very small with respect to the mechanical compliance of the syringes [18], and therefore the mechanical compliance of the catheter is neglected in our model. As a result, the flow rate entering the tube at time *t* and the flow rate leaving the tube (entering the blood stream) at the same time *t* are both equal to $u_{cath}(t)$, disregarding the different constitutions that the fluids entering, and leaving, respectively, may have in terms of the partial fluids R, G, and B.

Secondly, our new analytical method is introduced (see Fig. 3b), which enables incorporation of the “memory effect” of the catheter into the model. In order to make the analytical approach possible throughout the entire calculation up to the end of point (d) in the figure, a formulation in the Z-domain (using the Z-transform, which is a discrete variant of the Laplace transform) is introduced. This analytical method uses the results form the standard (Laplace-based) method [see (a)] as an input. This input has the form of general expressions for the various total flow rates as function of time. The Z-transform formulation in our method [see (b)] therefore does *not* replace the Laplace-based method from (a), but is used after it.

Third, the Z-domain formulation from (b) enables an easy, analytical, incorporation of the Poiseuille mixing effect (c) into the model, because the convolution-like nature of the Poiseuille mixing effect is reduced to a mere multiplication in the Z-domain. The mathematical details of the Poiseuille mixing effect are described in “Poiseuille mixing effect” section.

Finally (d), we derive expressions for the volume, and the first and second moment, of the dosing error as function of hardware parameters. These moments can be calculated directly within the Z-domain. From the first and second moment, key characteristics concerning timing and duration of the dosing error are derived.

Throughout the entire process, we have used the symbolic calculation capabilities of the Mathematica package (Mathematica 10, Wolfram^®^ Inc., USA) to process Laplace-transforms and Z-transforms analytically, as can be seen in “Results” section.

### Starting point: Laplace transform and Kirchhoff’s laws

In this paper, we focus on the multi-infusion of aqueous solutions, in which all solutions have a viscosity close to the viscosity of water. Therefore, in our mathematical model, all fluids are assumed to have approximately the same viscosity. For the calculation of the pressures and total flow rates, we use an electric circuit model that is analogous to the infusion set-up, in which current sources (the infusion pumps), resistances (infusion lines and catheter), and capacitors (mechanical compliance of the syringes) are present, and in which Kirchhoff’s laws are applied on the voltages (pressures) and currents (flow rates) in the Laplace domain. The output $U_{cath}(s)$ of the calculation is the Laplace transform of the total flow rate $u_{cath}(t)$ inside the catheter. This $u_{cath}(t)$ equals the total flow rate entering the tube at time *t* as well as the total flow rate leaving the tube (entering the blood stream) at the same time *t*. Let $\Gamma$ denote the complete set of these changes in pump flow rate setting values, together with the physical hardware parameters of the set-up, which are present in the form of explicit parameters in the analytical expressions for $U_{cath}(s)$. Using the Mathematica package (Mathematica 10, Wolfram^®^ Inc, USA), we were able to perform an inverse Laplace transform in order to retrieve an analytical expression for $u_{cath}(t, \Gamma )$ from the $U_{cath}(s, \Gamma )$, in which the above mentioned hardware parameters $\Gamma$ are present as explicit variables. This result is rendered in Eq. (33) in “Results” section.

### Key parameters of dosing errors

The dosing errors that are produced after changes in pump flow rate setting values in a multi-infusion set-up are of a temporary nature, i.e. “dead volume” and “mechanical compliance” (points (i) and (ii), respectively, as mentioned in the introduction) give rise to only a *temporary* deviation of the actual dose rates (entering the blood stream) from the intended ones (see e.g. Fig. 2). As a result, each of these dosing errors has the shape of a “bolus”.

Let $\beta ^{(R)}_{patient}(t)$ denote such a bolus-shapes dosing error, i.e. let $\beta _{patient}^{(R)}(t)$ denote the deviation from the intended partial flow rate [in this case of solution (R)], as a function of time, in which

6 $$\begin{aligned} \beta _{patient}^{(R)}(t) = u_{patient}^{(R)}(t) - u_{pump}^{(R)} \end{aligned}$$

Defining $t=0$ as the moment that a change in pump flow rate setting value takes place, and defining $\beta _{patient}^{(R)}(t)$ as the dosing error due to (only) this specific change in pump flow rate setting value at $t=0$, then we have by definition:

7 $$\begin{aligned} \forall t<0 : \beta _{patient}^{(R)}(t) = 0,  \quad\text{and} \quad \lim _{t\rightarrow \infty }\beta _{patient}^{(R)}(t) = 0 \end{aligned}$$

in agreement with the bolus-shaped nature of the dosing error $\beta _{patient}^{(R)}(t)$.

There are two basic mechanisms that contribute to the dosing errors, and the effect of each of these two mechanisms is restricted to a particular time interval: During the time interval $0< t < t_{delay}$, the dosing error entering the blood stream is caused by the push-out effect, as explained in Fig. 2. During the time interval $t > t_{delay}$, however, the dosing error entering the blood stream is caused by the effects of mechanical compliance, because the “push-out effect” is by definition restricted to the time interval $0< t < t_{delay}$. The effects of mechanical compliance are particularly strong directly after changing the pump flow rate settings, and thus the effects of mechanical compliance (in the form of deviating mixtures) are entering the catheter at point $\mathcal {M}$ at $t=0$, and hence entering the patient at point $\mathcal {P}$ directly after $t = t_{delay}$.

In the following, we will use $t^{POIS}_{delay}$ instead of $t_{delay}$, because, as will be demonstrated below, the Poiseuille profile of the flow affects the $t_{delay}$, resulting in a modified delay time that we will refer to as $t^{POIS}_{delay}$. Furthermore, we will consider a situation with two pumps (“Red” and “Green”) only, and will change the flow rate setting of the “Green” pump only (at $t=0$), leaving the flow rate setting of the “Red” pump (denoted as $u_{pump}^{(R)}$) constant. This does not affect the applicability of our method in more general cases. The flow rate setting of the “Green” pump changes from $u_{old}^{(G)}$ to $u_{final}^{(G)}$ at $t=0$.

The $\beta _{patient}^{(R)}(t)$ for $0< t < t^{POIS}_{delay}$ is due to the push-out effect, and hence its calculation is relatively easy. The $\beta _{patient}^{(R)}(t)$ for $t > t^{POIS}_{delay}$ however is due to the mechanical compliance, and hence its calculation is much more complex. Therefore, we split the $\beta _{patient}^{(R)}(t)$ into two parts according to the two corresponding distinct time intervals:

8 $$\begin{aligned} \beta _{patient}^{(R)}(t) = \left\{ \begin{array}{ll} 0 &{}\quad \text{ if }\; t< 0\\ \beta _{pushout}^{(R)}(t) = u_{pump}^{(R)} {{u_{final}^{(G)} - u_{old}^{(G)}}\over {u_{old}^{(G)} + u_{pump}^{(R)}}} &{} \quad \text{ if }\; 0< t< t^{POIS}_{delay}\\ \psi _{patient}^{(R)}(\tau ) \; \left( u_{final}^{(G)} + u_{pump}^{(R)} \right) &{} \quad \text{ if }\; t^{POIS}_{delay} < t. \end{array} \right. \end{aligned}$$

in which $\tau = t - t^{POIS}_{delay}$ and $\psi _{patient}^{(R)}(\tau )$ represents the effects of mechanical compliance for $t > t^{POIS}_{delay}$, and in which the dimensionless $\psi _{patient}^{(R)}(\tau )$ is bolus-shaped of its own:

9 $$\begin{aligned} \psi _{patient}^{(R)}(\tau ) = 0 \quad \text{ if }\; \tau < 0 \wedge \lim _{\tau \rightarrow \infty }\psi _{patient}^{(R)}(\tau ) = 0 \end{aligned}$$

In the following, we concentrate on finding analytical expressions for (the moments of) $\psi _{patient}^{(R)}(\tau )$. To this end, the discretized version $\psi _k^{(R)patient}$ of $\psi _{patient}^{(R)}(\tau )$ is defined as:

10 $$\begin{aligned} \psi _{patient}^{(R)}(\tau ) = \psi _{k^{*}}^{(R)patient}\quad \text{ with }\; k^{*} = \tau \, \left( u_{final}^{(G)} + u_{pump}^{(R)} \right) \, {{L}\over {\gamma V_{cath}}} \end{aligned}$$

### Contents of the catheter without poiseuille mixing effect

In this subsection, we derive a generic expression for the Z-Transform of the contents $\{a_k^{(R)}\}$ of the catheter, without incorporating the Poiseuille mixing effect. The Poiseuille mixing effect will be incorporated in the model later on.

First, let $\lambda _{tot}(t)$ denote the distance that the fluid inside the catheter has traveled as a result of the total flow rate $u_{cath}$, i.e.:

11 $$\begin{aligned} \lambda _{tot}(t) = {{L}\over {V_{cath} }}\, \int _0^{t} \; u_{cath}(t') \; dt' \end{aligned}$$

Furthermore, let $u_{\mathcal {M}}^{(R)}$ denote the actual partial flow rate of the Red fluid entering the catheter at the mixing point $\mathcal {M}$.

For some voxel *k* inside the catheter, the value of the ratio $a_k^{(R)}$ reflects the $u_{\mathcal {M}}^{(R)}(t^{*})$ divided by the total flow rate $u_{cath}(t^{*})$, at the time $t^{*}$ that the contents of that particular voxel entered the catheter at $\mathcal {M}$, i.e.:

12 $$\begin{aligned} a_k^{(R)} = {{u_{\mathcal {M}}^{(R)}(t^{*})}\over {u_{cath}(t^{*})}} \quad \text{ in } \text{ which } \;t^{*} \text{ is } \text{ such } \text{ that } \gamma k = \lambda (t^{*}) \end{aligned}$$

in which $\gamma = L/N$. In order to facilitate a Z-transform, we rewrite Eq. (12) in the following form:

13 $$\begin{aligned} a_k^{(R)} = {{L}\over {V_{cath} }} \, \int _0^{\infty } dt \; u_{\mathcal {M}}^{(R)}(t) \delta (\gamma k - \lambda _{tot}(t)) \end{aligned}$$

The value of the Dirac delta $\delta (\gamma k - \lambda _{tot}(t))$ is zero everywhere, except around $t = t^{*}$; hence evaluation of the integral in Eq. (13) shows the equivalence of Eqs. (12) and (13):

14 $$\begin{aligned}&{{L}\over {V_{cath} }} \, \int _0^{\infty } dt \; u_{\mathcal {M}}^{(R)}(t) \delta (\gamma k - \lambda _{tot}(t)) = {{L}\over {V_{cath} }} \, \int _{t^{*} - \varepsilon }^{t^{*} + \varepsilon } dt \; u_{\mathcal {M}}^{(R)}(t) \delta (\gamma k - \lambda _{tot}(t)) \nonumber \\&\quad \mathop {=}\limits ^{\varepsilon \rightarrow 0} \; {{L}\over {V_{cath} }} \, u_{\mathcal {M}}^{(R)}(t^{*}) \int _{t^{*} - \varepsilon }^{t^{*} + \varepsilon } dt \delta (\gamma k - \lambda _{tot}(t)) = {{L}\over {V_{cath} }} \, u_{\mathcal {M}}^{(R)}(t^{*})\int _{\gamma k-\eta }^{\gamma k+\eta } \; d\lambda \left( {{dt}\over {d\lambda }} \right) \delta (\gamma k-\lambda )\nonumber \\&\quad \mathop {=}\limits ^{\eta \rightarrow 0} \; {{L}\over {V_{cath} }} \, {{u_{\mathcal {M}}^{(R)}(t^{*})}\over {\left( {{d\lambda }\over {dt}} \right) _{t=t^{*}}}} = {{u_{\mathcal {M}}^{(R)}(t^{*})}\over {u_{cath}(t^{*})}} \end{aligned}$$

in which $\varepsilon$ and $\eta$ are both very close to zero.

Using the displacement rule from Z-transform theory [17], Eq. (13) now yields the following expression A(z) in the Z-domain:

15 $$\begin{aligned} A(z) = {{L}\over {{\gamma}V_{cath} }} \, \int _0^{\infty } dt \; u_{\mathcal {M}}^{(R)}(t) \, z^{-\lambda _{tot}(t)/{\gamma }} \end{aligned}$$

Here it needs to be mentioned that, since the Z-transform is merely a discrete form of the Laplace transform, it would be, theoretically, possible to replace this Z-transform by yet another Laplace transform. However, for clarity, we have chosen to use the Z-transform, because it connects to the “voxel-based” description of the contents inside the catheter in an intuitive way, and because it creates a clear distinction from the standard Laplace method used to calculate the pressures and flows that enter the mixing point $\mathcal {M}$.

Similarly, we may define the basic input for the calculation of the dosing error of fluid (R) as the difference between $u_{\mathcal {M}}^{(R)}$ (i.e. the actual partial flow rate of the Red fluid entering the catheter at the mixing point $\mathcal {M}$), and the intended $u_{pump}^{(R)}$ (i.e. the intended partial flow rate of the Red fluid entering the catheter at the mixing point $\mathcal {M}$):

16 $$\begin{aligned} u_{\mathcal {M}}^{(R)diff} = u_{\mathcal {M}}^{(R)} - u_{pump}^{(R)} \end{aligned}$$

yielding

17 $$\begin{aligned} A^{diff}(z) = {{L}\over {{\gamma}V_{cath} }} \, \int _0^{\infty } dt \; u_{\mathcal {M}}^{(R)diff}(t) \, z^{-\lambda _{tot}(t)/{\gamma }} \end{aligned}$$

This approach now enables easy incorporation of the Poiseuille mixing effect into our model using a simple multiplication in the Z-domain, as is explained below.

### Poiseuille mixing effect

**Fig. 4:**
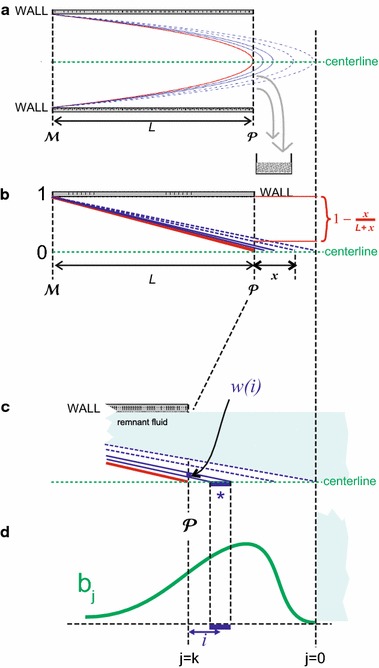
Schematic representation of the Poiseuille mixing effect, as explained in the text. **a** Poiseuille flow profile. **b** Linear relation between concentration and distance. **c** Geometry underlying the weight factor *w(i)*. **d** Convolution with green curve

The basic input for calculation of the Poiseuille mixing effect is the input from the Red line at $\mathcal {M}$ in the form of

18 $$\begin{aligned} a_{k=N}^{(R)diff}(t) = {{u_{\mathcal {M}}^{(R)diff}}(t)\over {u_{cath}}} \end{aligned}$$

which corresponds to $A^{diff}(z)$ in the z-domain [see Eq. (17)]. The mixing effect due to the Poiseuille profile causes a spreading out of the dosing error in time, in which the first arrival of the dosing error occurs sooner then it would without Poiseuille mixing effect. In a Poiseuile flow, the velocity along the centerline of the tube equals two times the average velocity. This implies that, after a change in pump flow rate settings at $t=0$, the tip of the parabolic flow profile reaches point $\mathcal {P}$ at the end of the catheter already at $t = t_{delay}/2$. Hence, we define $t^{POIS}_{delay}$ as: $t^{POIS}_{delay} = t_{delay}/2$. Because the Poiseuille mixing effect is a phenomenon that is created within the catheter during the very passage of the liquid through the catheter from point $\mathcal {M}$ to point $\mathcal {P}$, an accurate description of the full extent of this effect can only be given in the form of the contents of the very last voxel at point $\mathcal {P}$, just before this contents is released into bloodstream of the patient. This is illustrated in Fig. 4, in which the top diagram (Fig. 4a) shows the Poiseuille profile along the length of the catheter from point $\mathcal {M}$ to point $\mathcal {P}$, in which the tip of the red, innermost, parabola has just finished its journey through the catheter, and is just to be released into the blood stream. Each parabola in Fig. 4a constitutes a boundary between two volumes of liquid that once were subsequent, undistorted, “regular” voxels when they entered the catheter at $\mathcal {M}$. These initially flat boundaries between voxels near $\mathcal {M}$ are being stretched and distorted into the parabola-shaped boundaries during their travel to $\mathcal {P}$.

In “Appendix”, it is derived that the Poiseuille flow profile causes a mixing effect that features a simple linear relation between concentration and distance along the line from $\mathcal {M}$ to $\mathcal {P}$ (see Fig. 4b), which is consistent with earlier work by e.g. Taylor [19] and Hutton and Thornberry [20].

Using this simple linear relationship, the constitution of the final voxel of the catheter at $\mathcal {P}$ can be defined in terms of the original, undistorted, voxels that once entered the catheter at $\mathcal {M}$, as is illustrated in Fig. 4c. For instance, the contents of the “voxel $*$”, indicated by the symbol $*$, which is voxel nr *i* counting from point $\mathcal {P}$, and which is limited by two downward sloping lines and the thick blue horizontal line segment in Fig. 4c, contributes to the final voxel at $\mathcal {P}$ with weight factor $w_i$ (indicated by the small vertical thick blue line segment). The dimensionless weight factor $w_i$ equals the voxel length $\gamma$ times the first derivative of the expression ${{x}\over {L + x}}$, which is depicted in Fig. 4b, and which is established on the basis of geometrical reasoning using the triangles involved:

19 $$\begin{aligned} w_i = \gamma \, \left[ {{d}\over {dx}}\left( {{x}\over {L+x}} \right) \right] _{x=\gamma i} \end{aligned}$$

which yields

20 $$\begin{aligned} w_i = \frac{\gamma L}{(\gamma i + L)^2} \end{aligned}$$

Furthermore, the contents of each “voxel” *i*, with *i* running from 0 to infinity, equals that of voxel $b^{(R)}_{k-i}$, in which $b^{(R)}$ refers to the green curve in Fig. 4d, which is a “stretched-out” version of the original voxels $a^{(R)diff}_j$ that entered the catheter at $\mathcal {M}$, i.e.:

21 $$\begin{aligned} b_{2j}^{(R)} = a^{(R)diff}_j \end{aligned}$$

which reflects the fact that the velocity along the centerline of the tube equals two times the average velocity. As a result, the summation of all contributions $b^{(R)}_{k-i}$ with weight factors $w_i$ yields a (discrete) convolution, which is visible in the first term of the following equation, in which the fraction of the Red solution inside the last voxel at point $\mathcal {P}$ is denoted by the dimensionless scalar $\psi _k^{(R)patient}$, and in which *k* refers to the position of the beginning of the green curve *b* (which shifts in time):

22 $$\begin{aligned} \psi _k^{(R)patient} = \underbrace{\sum _{j=0}^k b_{k-j}^{(R)} w_{j}}_{\text{ convolution } \text{ due } \text{ to } \text{ poiseuille }} + \underbrace{{{L}\over {(L + \gamma k)}}\frac{ u_{final}^{(R)} \left( u_{final}^{(G)}-u_ {old}^{(G)}\right) }{ \left( u_{final}^{(G)}+u_{final}^{(R)}\right) \left( u_ {old}^{(G)}+u_{final}^{(R)}\right) }}_{\text{ remnant } \text{ fluid } \text{ from } \text{ old } \text{ mixture } \text{ due } \text{ to } \text{ poiseuille }} \end{aligned}$$

The second term in Eq. (22) does not depend on the compliances and resistances in the multi-infusion set-up, but represents the remnant fluid from the old mixture due to the poiseuille flow profile.

**Fig. 5:**
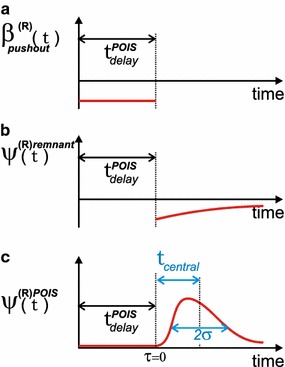
Sketch of key characteristics of a dosing error distribution. The $t_{central}$ and $\sigma$ are derived from the first and second moment, respectively, calculated directly in the Z-domain, *without the need* for an *inverse* Z-transform, as is explained in the text. **a** Push-out effect before the tip of Poiseuille profile has reached the patient. **b** Decaying remnant of push-out effect, due to Poiseuille mixing. **c** Dosing error due to mechanical compliances

When calculating the first and second moment of $\psi _k^{(R)patient}$ (or $t_{central}$ and the variance $\sigma ^2$ from Fig. 5, respectively), these moments are simply the sum of the moments of the two distinct terms in Eq. (22). The moments of the second term can be calculated directly in the spatial domain (using an upper limit to the amount of fluid). For the first term, however, the Z-transform is needed, as will be explained below. Therefore, we split the $\psi _k^{(R)patient}$ according to:

23 $$\psi _k^{(R)patient} = \psi _k^{(R)POIS} + \psi _k^{(R)remnant} $$

in which $\psi _k^{(R)POIS} = \sum _{j=0}^k b_{k-j}^{(R)} w_{j} \; \text{ and } \; \psi _k^{(R)remnant} = {{L}\over {(L + \gamma k)}}\frac{ u_{pump}^{(R)} (u_{final}^{(G)}-u_ {old}^{(G)})}{ \left( u_{final}^{(G)}+u_{pump}^{(R)}\right) \left( u_ {old}^{(G)}+u_{pump}^{(R)}\right) }$ and from now on will concentrate on the calculation of the first term, i.e. $\psi _k^{(R)POIS}$, only.

Performing the Z-transform on $\psi _k^{(R)POIS}$ yields:

24 $$\begin{aligned} \sum _{j=0}^k b_{k-j}^{(R)} w_{j}\, \mathop {\longmapsto }\limits ^{{{\mathcal {Z}}}{transform} } \, B(z) W(z) \end{aligned}$$

in which *B*(*z*) is the Z-transform of $b_j$, and *W*(*z*) is the Z-transform of $w_j$, which yields:

25 $$\begin{aligned} \gamma W(z) = \gamma - L (z-1) \left( \frac{1}{z} + \frac{2^{-L/{\gamma }}}{z^2}+\frac{3^{-L/{\gamma }}}{z^3}+\frac{4^{-L/{\gamma }}}{z^4}+\frac{5^{-L/{\gamma }}}{ z^5}+ \cdots \right) \end{aligned}$$

Since $L/{\gamma } = N$ is a large number, this constitutes a rapidly converging series for $z \approx 1$. Furthermore, since $b_{2j}^{(R)} = a_j^{(R)diff}$ (Eq. 21), we have $B(z)=A^{diff}(z^2)$, in which $A^{diff}(z)$ is the Z-transform of the original $a_k^{(R)diff}$, in the form in which it entered the catheter at $\mathcal {M}$, before being distorted due to the Poiseuille mixing effect [see Eq. (17)].

Therefore, in summary, we now have

26 $$\begin{aligned} \Psi ^{(R)POIS}(z) = A^{diff}(z^2) W(z) \end{aligned}$$

in which $\Psi ^{(R)POIS}(z)$ is the Z-transform of $\psi _k^{(R)POIS}$. This expression for $\Psi ^{(R)POIS}(z)$, i.e. Eq. (26), constitutes the central equation in our method, and enables derivation of explicit expressions for the first and second moment of $\psi _k^{(R)POIS}$ (or $t_{central}$ and $\sigma$ from Fig. 5, respectively), *without the need* of performing an *inverse* Z-Transform.

### Calculation of moments of the dosing error distribution after T$_{delay}$

We will now use the “theorems of moments” from Z-transform theory to provide expressions for key characteristics of the $\psi _{patient}^{(R)}(\tau )$ that enters the patient.

The zero’th moment yields:

27 $$\begin{aligned} \Lambda _0 = {{L}\over {{\gamma}V_{cath}}} \int _0^{\infty } \, \left( u_{\mathcal {M}}^{(R)}(\tau ) - u_{pump}^{(R)}\right) \, d\tau \end{aligned}$$

and hence the total volume of the dosing error $\psi _{patient}^{(R)}(\tau )$ equals:

28 $$\begin{aligned} Q = \int _0^{\infty } \, \left( u_{\mathcal {M}}^{(R)}(\tau ) - u_{pump}^{(R)}\right) \, d\tau \end{aligned}$$

The first moment yields:

29 $$\begin{aligned} \mu = -\, \lim _{z\rightarrow 1}\left( z {{d}\over {dz}}\Psi ^{(R)POIS}(z) \right) \end{aligned}$$

and hence

30 $$\begin{aligned} t_{central} = - {{V_{cath}}\over {\Lambda _{0} u_{final}}}\lim _{z\rightarrow 1} \left( z {{d}\over {dz}}\Psi ^{(R)POIS}(z)\right) \end{aligned}$$

Furthermore, using the second moment, the following general expression for $\sigma$ is obtained, in which the typical “duration” or “width” of a dosing error is characterized as $2\sigma$ (see Fig. 5):

31 $$\begin{aligned} \sigma = {{V_{cath}}\over {u_{final}}}\sqrt{{{1}\over {\Lambda _0}} \lim _{z \rightarrow 1} \, \, \left( z^2 {{d^2}\over {dz^2}}\Psi ^{(R)POIS}(z) + z {{d}\over {dz}}\Psi ^{(R)POIS}(z) \right) - \left( {{\mu }\over {\Lambda _0}}\right) ^2 } \end{aligned}$$

In “Results” section, Eqs. (27)–(31) will be used to produce analytical expressions for various situations. Furthermore, also in “Results” section, Eqs. (27)–(31) will be evaluated for situations *without* the Poiseuille mixing effect, in which case the $\Psi ^{(R)POIS}(z)$ in these equations is replaced by merely *A*(*z*).

### Spectrometric in-vitro set-up for feasibility tests

**Fig. 6:**
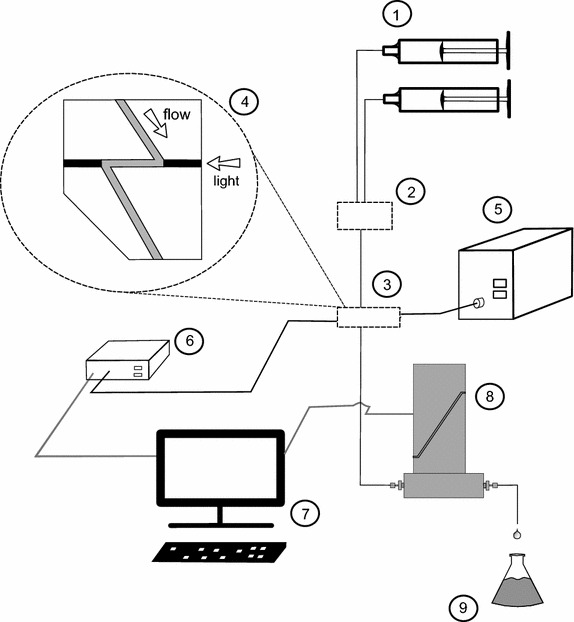
Experimental setup: the syringe pumps (*1*) pump their contents towards a mixing point (*2*). From the mixing point, the fluid will flow towards a flow cell (*3*), (*4*), where the light from light source (*5*) is used for an absorbance measurement by the spectrometer (*6*). A computer will simultaneously read out the flow rates from the flow meter (*8*). Finally the fluid is stored inside a container (*9*)

A schematic of the in-vitro set-up is rendered in Fig. 6. Two Perfusor B. Braun^®^ syringe pumps (B. Braun, Melsungen AG, Germany) and 50-ml syringes (Melsungen AG, Germany) were used in the experimental setup. The syringes contained Tartrazine (TT) and Indigo Carmine (IC) solution, solved in distilled water. Each syringe was connected to an infusion line (d = 1 mm) of 200 cm (Cair LGL^®^, France) and subsequently combined using the using a 3-needle-free Y-connector (20038E7D, Cardinal Health^®^, Switzerland). The Y-connector outflow was connected to a flowcell (Z flowcell w/SMA 905, 10-mm pathlength, FIAlab^®^, Seattle, WA, USA), this flowcell was also connected to a visual light spectrum (250–2500 nm) DT-1000 light source (Ocean Optics^®^, Dunedin, FL, USA). This allowed a spectrometer QE65000 (Ocean Optics^®^, Dunedin, FL, USA), also connected to the same flowcell, to continuously measure an absorption spectrum of the dye mixture. Concentrations of each dye were acquired from this absorption spectrum [4]. After the flow cell, the cumulative flow rate was measured using three M12p flowmeters (Bronkhorst^®^, Ruurlo, The Netherlands). Sample time of all the measurements was 1 s.

## Results

### General result from laplace transform as a starting point

**Fig. 7:**
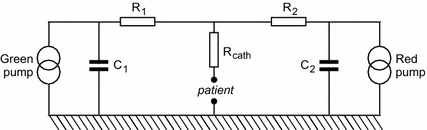
Electric analog of a multi-infusion setup with two pumps. See “Method: analytical model, and in-vitro set-up” section

Performing the first step in the scheme rendered in Fig. 3, a general expression for $u_{\mathcal {M}}^{(R)diff}$ has been derived in the Laplace domain, using a known technique, i.e. Laplace Transform in combination with Kirchhoff laws, on the basis of the electric analog depicted in Fig. 7:

32 $$\begin{aligned} \breve{u}_{\mathcal {M}}^{(R)diff}(s) = - \; \frac{ C_2 u_{downstep}^{(G)} R_{cath}}{s^2 \left( C_1 C_2 R_{cath} R_1+ C_1 C_2 R_{cath} R_2+ C_1 C_2 R_1 R_2\right) +s \left( C_1 R_{cath}+ C_1 R_1+ C_2 R_{cath}+ C_2 R_2\right) +1} \end{aligned}$$

in which $\breve{u}_{\mathcal {M}}^{(R)diff}(s)$ is the Laplace transform of $u_{\mathcal {M}}^{(R)diff}(t)$, and $u_{downstep}^{(G)}$ equals the change in pump flow rate setting of the Green pump at $t=0$, and $C_1$ and $C_2$ are the mechanical compliances of the “green” and “red” syringe, respectively, and $R_1$ and $R_2$ the resistances of the “green” and “red” feeding lines, respectively. Meanwhile, the pump flow rate setting of the Red pump, $u_{pump}^{(R)}$ remains unchanged all the time. Using the inverse Laplace transform function of Mathematica, the following general expression was obtained in the time domain:

33 $$\begin{aligned} u_{\mathcal {M}}^{(R)diff}(t) = \frac{\left( e^{-\frac{t}{\vartheta _{first}}} - e^{-\frac{t}{\vartheta _{second}}}\right) u_{downstep}^{(G)} R_{cath} C_2 }{ \sqrt{b^2-4 a c}} \end{aligned}$$

in which

34 $$\begin{aligned} \vartheta _{first}&=   {{1}\over {2}}\left( b - \sqrt{b^2 - 4 a c}\right) \nonumber \\ \vartheta _{second}&= {{1}\over {2}}\left( b + \sqrt{b^2 - 4 a c}\right) \nonumber \\ a&= R_{cath} R_1 + R_{cath} R_2 + R_1 R_2 \nonumber \\ b&= C_1(R_{cath}+R_1) + C_2(R_{cath}+R_2) \nonumber \\ c&= C_1 C_2 \end{aligned}$$

Using Eq. (32), the total volume of the dosing error [i.e., the time-integral of Eq. (33)] is easily calculated using the general rules for integration and for calculation of limits from Laplace transform theory. The resulting expression $Q^{(R)}_{dosingerror}(\Gamma )$, i.e. the volume of the dosing error, depends on the set of hardware parameters $\Gamma$. In this specific case, the parameter set $\Gamma$ comprises of: $\Gamma = \{Resistances, Compliances, Flow Rates\}$.

35 $$\begin{aligned} Q^{(R)}_{dosingerror}(\Gamma ) = C_2 R_{cath} u^{(G)}_{downstep} \end{aligned}$$

**Fig. 8:**
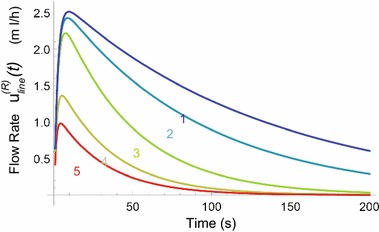
Flow rate $u_{\mathcal {M}}^{(R)diff}(\tau )$ as function of time $\tau$ for different values of the resistance R$_{cath}$ and the mechanical compliance C$_{red}$, according to the theoretical model. (*1*) $C_{red}=C_{standard}, R_{cath}=3 R_{standard}.$ (*2*) $C_{red}=C_{standard}, R_{cath}=2 R_{standard}.$ (*3*) $C_{red}=C_{standard}, R_{cath}=R_{standard}$. (*4*) $C_{red}= 0.52 C_{standard}, R_{cath}=R_{standard}$. (*5*) $C_{red}= 0.36 C_{standard}, R_{cath}=R_{standard}$

The subtraction of two exponentially decaying functions in Eq. (33) yields a characteristic bulb-shaped graph of $u_{\mathcal {M}}^{(R)diff}(\tau )$ as function of the time $\tau$; see Fig. 8. In this figure, Eq. (33) has been evaluated for a number of parameter settings of $\Gamma$.

From the general equation, more specific and practical expressions can be derived by substituting standard values into the equation, leaving the parameters of interest as variables. The standard values for $R_{cath}$, $R_1$, $R_2$, $C_1$, $C_2$, $u_{pump}^{(R)}$, $u_{old}^{(G)}$ and $u^{(G)}_{downstep}$ are rendered in Table 1.

**Table 1:** Standard values in simulations and in-vitro measurements

Variable	Standard value
$R_{cath}$	1145 Pa/(ml/h)
$R_1$	23 Pa/(ml/h)
$R_2$	23 Pa/(ml/h)
$C_1$	1.5 10$^{-5} \;$ ml/Pa
$C_2$	1.5 10$^{-5} \;$ ml/Pa
$u_{pump}^{(R)}$	0.5 ml/h
$u_{old}^{(G)}$	12 ml/h
$u^{(G)}_{downstep}$	6 ml/h

### Typical example: resulting dosing error during syringe exchange

We now consider the specific case in which the “Green” syringe is exchanged, during which the green pump is stopped and green line has been clamped (with a Kocher), but the red line is not clamped, and the settings of the red pump remain unchanged. Let $T_{restart}$ denote the time between clamping the green line and the reopening the green line, i.e. $T_{restart}$ denotes the time duration of the entire procedure of exchanging the green syringe.

**Fig. 9:**
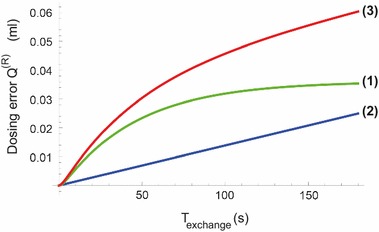
Calculated total dosing error of the Red fluid as function of the time duration $T_{exchange}$ of the syringe exchange procedure, in which the green line has been fully closed (clamped) during the entire syringe exchange procedure, and in which $C_{red}=C_{standard}, R_{cath}=R_{standard}$. (*1*) Compliance effect only (see Eq. 36). (*2*) “Red pump on” effect only. (*3*) Compliance and “red pump on” effects combined

During the syringe exchange time interval, the red line still oozes red fluid in undiluted form into the first voxels of the catheter directly after the mixing point $\mathcal {M}$. We now consider two scenario’s within the general scenario of the syringe exchange with the green line being clamped off during the entire exchange time interval. In the first scenario, the red line is not clamped off, but the red pump is switched off (pump flow rate setting value is zero; still connected to the red line) simultaneously at the beginning of the syringe exchange procedure, and restarted to its original pump flow rate setting value at the very end of the syringe exchange procedure. In the second scenario, no actions are performed in relation to the red pump or red line at all. The first scenario entails that accumulation of undiluted red fluid directly beyond the mixing point is caused only by the compliance of the red syringe, in combination with the drop in pressure caused by the clamping of the green line. This is indicated by the text “compliance” under the second brace in Eq. 36. In the second scenario, however, the regular pumping action of the red pump (at flow rate $u_{pump}^{(R)}$) adds to the accumulation of undiluted red fluid beyond the mixing point as well (indicated by the text “red pump on” under the first underbrace in Eq. 36). As a result, in the second scenario, the total dosing error $Q^{(R)}_{dosingerror}(T_{exchange})$ is the sum of the “compliance” and the “red pump on” effects. See Fig. 9. After $t_{delay}$, this total dosing error is entering the blood stream of the patient within a very short time interval, due to the fact that the green pump has a high flow rate.

36 $$\begin{aligned} Q^{(R)}_{dosingerror}(T_{exchange}) = \underbrace{u_{pump}^{(R)} T_{exchange}}_{\text{ red } \text{ pump } \text{ on }} + \underbrace{ {{ u_{downstep}^{(G)} R_{cath} C_2\left( \vartheta _{first}\left( 1 - e^{-\frac{T_{exchange}}{\vartheta _{first}}} \right) - \vartheta _{second} \left( 1 - e^{-\frac{T_{exchange}}{\vartheta _{second}}} \right) \right) }\over { \sqrt{b^2-4 a c}}} }_{\text{ compliance }} \end{aligned}$$

In Eq. (36), the second term (“compliance”) has been calculated on the basis of integrating the right side of Eq. (33) over time.

### Analytical results for reducing the flow rate of the green pump, without Poiseuille mixing effect

We now consider the more general case in which the pump flow rate setting of green pump, i.e. of the fast pump, is lowered with an amount $u^{(G)}_{downstep}$, without clamping any line. The volume $Q^{(R)}_{dosingerror}$ does not depend on the specific distribution of the dosing error over time, and hence yields the same result as in Eq. (35).

Here we present the general result of the calculation of the first moment of A(z) without the Poiseuille flow effect:

37 $$\begin{aligned} t_{central} = {{V_{cath}}\over {L}} \; \frac{\int _0^{\infty } \lambda (t) u_R(t) \, dt}{u_{final} C_2 R_{cath} u^{(G)}_{downstep}} \end{aligned}$$

Applying this result to the specific case described in Eq. (32), i.e. lowering the pump flow rate setting of the green pump, and substituting Eq. (33) into Eq. (37), yields the following result:

38 $$\begin{aligned} t_{central} = b + \frac{u^{(G)}_{downstep} }{4 u_{final}}\left( \sqrt{b^2-4 a c} - 2 C_2 R_{cath} + b + 2 {{a c}\over {b}}\right) \end{aligned}$$

In many clinical situations we have $C_1 = C_2 = C$ and $R_1 = R_2 = R$, in which case Eq. (38) reduces to:

39 $$\begin{aligned} t_{central} = 2 C(R_{cath} + R) + \frac{C u^{(G)}_{downstep} \left( 2 R_{cath}^2 + 6 R_{cath} R + 3 R^2\right) }{4 u_{final} (R_{cath} + R)} \end{aligned}$$

Applying Eq. (31) from the “Method: analytical model, and in-vitro set-up” section to the present situation, without the Poiseuille flow effect reads:

40 $$\begin{aligned} \sigma = {{V_{cath}}\over {L}} \; {{1}\over {u_{final}}}\sqrt{\frac{\int _0^{\infty } \lambda (t)^2 u_R(t) \, dt}{C_2 R_{cath} u^{(G)}_{downstep}}-\frac{\left( \int _0^{\infty } \lambda (t) u_R(t) \, dt\right) {}^2}{\left( C_2 R_{cath} u^{(G)}_{downstep}\right) ^2}} \end{aligned}$$

Evaluation of Eq. (40) yields a very long and unwieldy expression. However, as has been noted before, in many clinical situations we have $C_1 = C_2 = C$ and $R_1 = R_2 = R$, and, furthermore, for most catheters we have $R \ll R_{cath}$. Applying these assumptions, we obtain the following result:

41 $$\begin{aligned} \sigma = {{C R_{cath}}\over {{2 \sqrt{3} \, \, u_{final}}}}\sqrt{ \left( u^{(G)}_{downstep}\right) ^2+12 u^{(G)}_{downstep} u_{final}+48 (u_{final})^2} \end{aligned}$$

This result will be compared with the findings from the in-vitro experiments in “Results of in-vitro experiments, and comparison with theoretical predictions” section.

If, in Eq. (41), the $u^{(G)}_{downstep}$ would be very small with respect to $u_{final}$, then this expression for $\sigma$ approaches the familiar $\sigma \approx 2 C R_{cath}$.

### Analytical results for reducing the flow rate of the green pump, incorporating the Poiseuille mixing effect

Evaluation of Eq. (31) from “Method: analytical model, and in-vitro set-up” section to the situation of reducing the pump flow rate setting of the green pump (still without any clamping) once more, but now substituting $\Psi ^{(R)POIS}(z)$ [instead of *A*(*z*)] in order to incorporate the Poiseuille flow effect, yields a very long and unwieldy expression. Therefore, again, we apply the assumptions $C_1 = C_2 = C$ and $R_1 = R_2 = R$ and $R \ll R_{cath}$, which yields the following result:

42 $$\begin{aligned} \sigma ^{POIS} = {{1}\over {{\sqrt{3} \, \, u_{final}}}}\sqrt{ C^2 R_{cath}^2 \left( \left(u^{(G)}_{downstep}\right)^2+12 u^{(G)}_{downstep} u_{final}+48 (u_{final})^2 \right) + 3 (V_{cath})^2 } \end{aligned}$$

As can be seen in Eq. (42), the strength of the contribution of the Poiseuille mixing effect to the width $\sigma ^{POIS}$ depends on the internal volume of the catheter $V_{cath}$ with respect to the time $C R_{cath}$ in combination with the flow rates $(u^{(G)}_{downstep})$ and $u_{final}$; or, more precisely, on the ratio between $3 (V_{cath})^2$ and $C^2 R_{cath}^2 ((u^{(G)}_{downstep})^2+12 u^{(G)}_{downstep} u_{final}+48 (u_{final})^2 )$.

If the catheter has a large internal volume but the time $C R_{cath}$ is short and the flow rates are low, then the $\sigma ^{POIS}$ in Eq. (42) reduces to: $\sigma ^{POIS} \approx V_{cath} / u_{final}$.

If, however, in Eq. (42), the $u^{(G)}_{downstep}$ would be very small with respect to $u_{final}$, and the time $C R_{cath}$ would be very large and the $V_{cath}$ would be small, then this expression for $\sigma$ approaches $\sigma ^{POIS} \approx 4 C R_{cath}$, which is two times larger than the $\sigma \approx 2 C R_{cath}$ that was calculated before when omitting the Poiseuille mixing effect. This factor two is a result from the fact that, in a Poiseuille flow, the velocity at the centerline of the catheter equals two times the average velocity.

### Results of in-vitro experiments, and comparison with theoretical predictions 

**Fig. 10:**
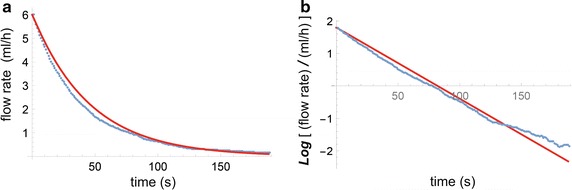
Results of in-vitro experiments, in which the flow rate of the Green fluid (*blue dots*) is plotted as function of time, after a change in the flow rate setting of the Green pump. The predicted theoretical result of the Green fluid flow is rendered by the *red line*. **a** Linear vertical axis. **b** Logarithmic vertical axis

**Fig. 11:**
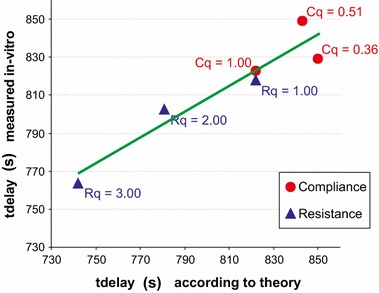
Results of in-vitro experiments, in which the measured tDelay (*red dots* and *blue triangles*) are plotted as function of the corresponding theoretical value. *Red dots* using a syringe of 50, 25 or 10 cc. *Blue triangles* using various resistances. *Green line* linear regression line. Pearson’s R was 0.95; standard deviation between theory and experiment was 16.2 s

**Fig. 12:**
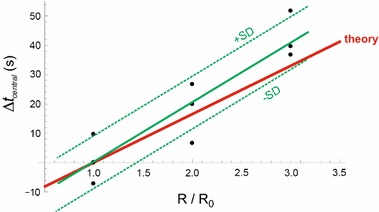
Comparison of theoretical results (*red solid line*) with the results in-vitro experiments (*black dots*), in which the measured $\Delta t_{central}$ is plotted as function of the value of the relative resistance R of the catheter with respect to its standard value $R_0$, in which $\Delta t_{central}$ is defined as the measured or calculated value of $t_{central}$ with respect to the standard value of $t_{central}$ as calculated for $R_0$. *Solid green line* Linear regression line. Pearson’s R was 0.89; standard deviation between theory and experiment was 8.4 s. *Dashed green lines* regression line ± standard deviation

Three types of in-vitro experiments have been performed, in order to compare theoretical results with actual measurements. These three types of experiments are:

(i) measurement of the flow rate of the Green fluid as function of time, immediately after a change in pump flow rate setting value of the green pump, corresponding to $$u^{(G)}_{downstep} = $$ 6 ml/h; see Fig. 10.
(ii) measurement of $t_{delay}$ for a set of three different syringes and three resistances, corresponding to various values of *C* and $R_{cath}$; see Fig. 11. The experimental measurement of the $t_{delay}$ showed a standard deviation between theory and experiment of 16.2 s, which is 14% of the total range of variations in $t_{delay}$.
(iii) measurement of $\Delta t_{central}$ as function of various values of the relative resistance of the catheter with respect to its standard value R0, in which $\Delta t_{central}$ was defined as the measured $t_{central}$ minus the standard value of $t_{central}$ for $R_{cath}=R0$. Three different values of $R_{cath}/R_0$ have been used; and the measurement $\Delta t_{central}$ of was repeated three times for each value of $R_{cath}/R_0$. See Fig. 12. The experiment measuring the $\Delta t_{central}$ showed a reasonable agreement between theory and measurements, in which standard deviation between theory and experiment was 8.4 s, which is 13% of the total range of variations in $\Delta t_{central}$.

## Discussion

In this paper, explicit expressions have been derived for the total volume (Q), central time point ($t_{central}$) and “width” or “duration” ($2\sigma$) of dosing errors as function of hardware parameters such as mechanical compliance of syringes, resistance of the catheter, length of the catheter, for two general cases of a change in pump flow rate setting value in a multi-infusion set-up, as well as for a typical case of syringe change-over. Consistency of the resulting analytical expressions has been examined for limiting cases, and, more importantly, three types of in-vitro measurements have been performed to obtain a first experimental test of the validity of the theoretical results derived in this paper. The experimental measurement of the $t_{delay}$ (Fig. 11) showed a standard deviation between theory and experiment of 16.2 s, which is 14% of the total range of variations in $t_{delay}$. The experiment measuring the $\Delta t_{central}$ (Fig. 12) showed a reasonable agreement between theory and measurements, in which standard deviation between theory and experiment was 8.4 s, which is 13% of the total range of variations in $\Delta t_{central}$.

### General findings from the expressions derived

In many of the resulting expressions as presented in “Results” section, the relative contribution of various factors affecting the dosing errors, such as the poiseuille mixing effect, can be discerned clearly in the structure of these expressions. The characteristic “time constant” $R_{cath} C$ is clearly recognisable in the expressions. Furthermore, the ratio between the $u^{(G)}_{downstep}$ (the size of the change in pump flow rate setting value) and the $u_{final}$ (the stabilized final flow rate) appears as an important factor in determining the nature of the dosing error bolus, particulary how the dosing error will spread out in time (the “width” or “duration” ($2\sigma$) of the dosing error bolus). The contribution of the Poiseuille mixing effect is visible in the resulting expressions. Of particular clinical importance is the fact that the Poiseuille flow profile, once fully developed, causes a significant reduction of the time that is needed for a newly administered medication to reach the patient. This may come as an unexpected effect for the clinician, as may also the fact that the value of $2\sigma$ (the “spread out”) is increased at the same time. The results in this paper may help to determine the magnitude of this “spread-out” effect as function of the hardware parameters (most notably, the characteristic $R_{cath} C$ time), and e.g. the length and resistance of the catheter.

### Limitations of the method, and possible extensions

A number of limitations can be identified in our method in its present form; most apparent is the assumption that when a time interval of duration $t^{POIS}_{delay}$ has lapsed after having changed a pump flow rate setting value, the actual flow rate has already stabilized and reached the value $u_{final}$. This needs not to be true in a general case. However, using the same reasoning as presented in this paper, our method can be extended to include non-stable flow rates at $t=t^{POIS}_{delay}$ as well. Another limitation may arise by the fact that in our method we did not examine other elements (other than just syringes, infusion lines, catheters, and pumps) that may be present in an infusion set-up, such as a non-return valve (preventing flow back from the catheter into a line towards a pump), or filters. The non-return valves may be incorporated into the method by restricting flow rates in the catheter to positive values or zero, whereas filters may be modeled on basis of a resistance and a mechanical compliance, as indicated in the literature (e.g. [21]). Another complicating factor may be the use of high viscosity fluids in the infusion set-up. In our method, as it stands now, all fluids in the system were assumed to be of the same viscosity. Incorporation of deviating (high) viscosities into our method would entail an extension of the laminar flow profile used in this paper, because differences in viscosity of mixing liquids may produce destabilization of laminar flow. An easy, but useful, extension of our method is the incorporation of the possibility that the *height* at which the syringes are positioned in a set-up near the patient, is altered during the period that a patient receives medication using the multi-infusion set-up. Since a change in height may be modeled as the addition of an extra pressure source within the model during the process of infusion, this results in an extra bolus of dosing error, and, hence, in the addition of some extra terms and factors in the equations in “Results” section. Finally, the parabolic flow profile of a laminar flow does not come into existence instantaneously at the mixing point; it has been calculated [20] how long, c.q. what distance, it takes for the flow profile to approach the parabolic shape. All of these complicating factors need to be incorporated into the model to make it more realistic. As far as we can anticipate now, we do not expect these extra factors to be incompatible with our general approach outlined in this paper; however, further research is needed.

### Potential use of the results in clinical practice

Healthcare professionals working with infusion technology in critical care have expressed the desire for a real-time tool that visualizes the multi-infusion drug therapy, e.g. continuously calculates predictions to indicate when the drugs will be entering the blood stream of the patient and in what dose. Such a tool may also visualize the causal consequences of an intervention (i.e., change in a pump flow rate setting value), before a clinician decides to proceed with such an intervention. In order to develop such a tool in the future, a fast and generic model will be necessary, combining all the relevant physical effects. The desirability of such a visualization tool, and the mathematical modeling that is a pre-requisite for the development of such a tool in the future, is what prompted us to go beyond the state-of-the-art and to develop the fully analytical method described in this paper. We envision three types of developments in which the results from this paper may be useful: (i) an interactive tool, running synchronized with the multi-infusion system on a smartphone device or on a bed-side display, e.g., next to the vital signs display monitors. Such a tool could then be used in several cases, such as inotropic titration, or to visualize the effects of a syringe changeover, or even the consequence of changing the height of a pump during infusion. (ii) Another application of the method presented in this paper could be actual computer control of an infusion system. It has been shown that a computer-controlled pump with “knowledge” about the dead volume and the mixing effect within the dead volume can be useful in preventing overshoot [16]. Moreover, a control system with a feedback approach has also been attempted, where the mean arterial blood pressure was used to control the administration of a fast-acting vasodilator [22, 23]. In both cases, however, increasingly complex situations and infusion setups were encountered where only the incorporation of all the interdependent physical effects, as described in this paper, would provide a sufficiently accurate prediction in order to make computer control feasible. (iii) The model can potentially also be used as an analysis and design tool, prior to investing in potential new infusion hardware. It is known that flow characteristics are influenced by valves [24, 25], syringes [26], infusion lines and catheters [27–29], and filters [21]. By using the method from this paper, benefits of these components can be compared against potential tradeoffs.

## Conclusions

We have developed a new method that combines mechanical compressibility (compliance) effects with poiseuille flow and push-out effects (“dead volume”) in one single mathematical framework for calculating dosing errors in multi-infusion set-ups.

In contrast to existing numerical methods, our method produces explicit expressions that indicate the mathematical dependencies of the dosing errors on hardware parameters and pump flow rate settings.

The results from the in-vitro experiments show a reasonable to good agreement between measurements and theoretical results.

The relative contribution of various factors affecting the dosing errors, such as the poiseuille mixing effect, resistance and internal volume of the catheter, mechanical compliance of the syringes and the various pump flow rate settings, can now be discerned clearly in the structure of the expressions generated by our method.

This enables insight and predictability in a large range of possible situations involving many variables and dependencies, which is potentially very useful for e.g. the development of a fast, bed-side tool “calculator” that provides the clinician with a precise prediction of dosing errors and delay times interactively for many scenario’s. The interactive nature of such a device has now been made feasible by the fact that, using our method, explicit expressions are available for these situations, as opposed to conventional time-consuming numerical simulations. Other potential applications of our method involve analysis and design tools for new infusion hardware, and interactive devices connected to the infusion hardware that counteract impending dosing errors using predictive calculations.

## Abbreviations

ICU: intensive care unit; OR: operation room; MABP: mean arterial blood pressure.

### List of symbols

$$V_{cath}$$
internal volume of the catheter
$$t_{delay}$$
time needed to travel through the catheter
$$u_{cath}$$
volumetric flow rate inside the catheter
$$\mathcal {M}$$
mixing point where fluid enters the catheter
$$\mathcal {P}$$
tip of catheter where fluid enters the blood stream
*L*: length of the catheter
*N*: large dimensionless number, representing the number of voxels inside the catheter
$$\gamma $$
L/N, i.e. length of a single voxel inside the catheter
$$t=0$$
point in time at which a change in pump flow rate setting value takes place
$$a_k^{(R)}$$
fraction of the volume of voxel *k* that contains “red” solution
$$u_{patient}^{(R)}(t)$$
partial flow rate of the “red” solution that enters the blood stream at time *t*
$$u_{pump}^{(R)}$$, $$u_{pump}^{(G)}$$
pump flow rate setting of the “red”, and “green”, pump, respectively
$$\Gamma $$
complete set of flow rate settings and physical hardware parameters
$$\beta _{patient}^{(R)}(t)$$
dosing error of “red” solution, i.e. deviation from intended partial flow rate, as function of *t*
$$t_{delay}^{POIS} = {{1}\over {2}}t{delay} $$
time needed for the *tip of the Poiseuille flow* to travel through the catheter *along the central longitudinal axis of the catheter*
$$u_{old}^{(G)}$$
pump flow rate setting of the “green” pump before $t=0$
$$u_{final}^{(G)}$$
pump flow rate setting of the “green” pump after $t=0$
$$\tau $$
$$t - t_{delay}^{POIS}$$
$$\beta _{pushout}^{(R)}(t)$$
$$\beta _{patient}^{(R)}(t)$$ for $$0< t < t_{delay}^{POIS}$$
$$\psi _{patient}^{(R)}(\tau )$$
$$\beta _{patient}^{(R)}(t) / (u_{final}^{(G)} + u_{pump}^{(R)} )$$ for $$t_{delay}^{POIS} < t$$
$$\lambda _{tot}(t)$$
distance that the fluid would have travelled *without* the Poiseille mixing effect
$$u_{\mathcal {M}}^{(R)}(t)$$
the actual partial flow rate of “red” fluid *entering* the catheter at the mixing point $\mathcal {M}$ at time *t*
$$u_{\mathcal {M}}^{(R)diff}(t)$$
$$u_{\mathcal {M}}^{(R)}(t) - u_{pump}^{(R)}$$
$$w_i$$
weight factor for contribution of original voxel nr *i* to the voxel at the tip $\mathcal {P}$
$$b_{2j}$$
$$a_j^{(R)diff}$$
$$\psi _k^{(R)patient}$$
discrete form of $\psi _{patient}^{(R)}(\tau )$
$$\psi _k^{(R)POIS}$$
part of $\psi _k^{(R)patient}$ that is calculated in the Z-domain
$$\Psi ^{(R)POIS}(z)$$
Z-transform of $$\psi _k^{(R)POIS}$$
*Q*: total volume of the $\psi _k^{(R)POIS}$-part of the dosing error
$$t_{central}$$
see Fig. 5c
$$\sigma $$
see Fig. 5c
$$R_{cath}$$
resistance of the catheter
$$R_1$$
resistance of the “green” feeding line
$$R_2$$
resistance of the “red” feeding line
$$C_1$$
mechanical compliance of the “green” syringe
$$C_2$$
mechanical compliance of the “red” syringe
$$u_{downstep}^{(G)}$$
change in pump flow rate setting of the “green” pump at $t=0$
$$\vartheta _{first}$$, $$\vartheta _{second}$$, *a*, *b*, *c*
see Eq. (34)

## Figures and Tables

**Fig. 13:**
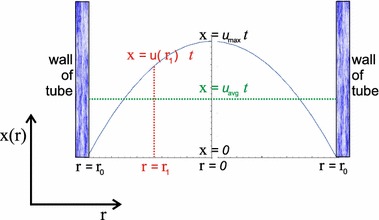
Poiseuille profile in laminar flow. See text

**Fig. 14:**
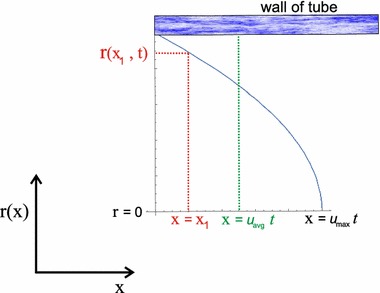
Same relation as in Fig. 13, but now with *r* as function of *x*. Effectively, this figure results from rotating Fig. 13 by 90°

## Appendix: Volume distribution in a Poiseuille flow

Laminar flow produces a Poiseuille flow profile, in which the velocity *u* depends on the distance *r* to the central longitudinal axis of the catheter [30]:

43 $$\begin{aligned} u(r) = {{1}\over {4\eta }} {{\Delta p}\over {L}} (r_0^2 - r^2) \end{aligned}$$

in which $\eta$ is the viscosity of the fluid, $\Delta p / L$ is the pressure drop over a length *L* of catheter, and $2r_0$ is the diameter of the catheter.

Furthermore, the average velocity $u_{avg}$ equals

44 $$\begin{aligned} u_{avg} = {{r_0^2 \Delta p}\over {8\eta L}} \end{aligned}$$

and the maximum velocity (located along the central longitudinal axis of the catheter) equals

45 $$\begin{aligned} u_{max} = 2 u_{avg} \end{aligned}$$

Evidently, if thermal diffusion is left out of the model and the fluid is considered to be completely homogeneous at the mixing point $\mathcal {M}$, then the length $\lambda _{max}(t)$ of the protrusion of the top of the Poiseuille profile into the catheter along the central longitudinal axis, is calculated easily by combining Eqs. (45) and (44):

46 $$\begin{aligned} \lambda _{max}(t) = t u_{max} = {{r_0^2 \Delta p}\over {4\eta L}} \, t \end{aligned}$$

Let *x* denote the position measured along the central axis along the catheter, starting withy $x=0$ at $\mathcal {M}$, and let $r_{boundary}(x,t)$ denote the distance with respect to the central longitudinal axis at position *x* at time *t*, at which the boundary (parabola) is situated. Multiplying both sides of Eq. (43) with *t*, yields the relation:

47 $$\begin{aligned} x\left( r_{boundary}\right) = {{1}\over {4\eta }} {{\Delta p}\over {L}} \left( r_0^2 - \left( r_{boundary}\right) ^2 \right) \,\, t \end{aligned}$$

in which *x* is a function of $r_{boundary}$ and represents the distance that the boundary (see Fig. 13) has travelled in the longitudinal direction (along the length of the catheter), for a given radial position $r_{boundary}$. The relation between x and r in Eq. (47) is depicted in Fig. 13:

Conversely, solving $r_{boundary}$ from Eq. (47) for all values $x \in [0, t \,\, u_{max}]$, we have:

48 $$\begin{aligned} r_{boundary}(x,t) = \sqrt{r_0^2 - {{4 x \eta }\over {t\left( {{\Delta p}\over {L}}\right) }} } \end{aligned}$$

which is depicted in Fig. 14.

In order to calculate the amount of the fluid *R* that is present inside a thin, disk-shaped, cross-sectional volume *S* of the catheter at position *x*, expressed as a fraction $f_R(x,t)$ of the total fluid in the thin volume *S*, in which the disk-shaped volume *S* is perpendicular to the central longitudinal axis of the catheter at point *x*, we need to integrate over the cross-sectional area of the catheter at point *x*, from $r=0$ to $r=r_{boundary}(x,t)$, and divide this by the total cross-sectional area $\pi r_0^2$:

49 $$\begin{aligned} f_R(x,t) = {{1}\over {\pi r_0^2}} \int _0^{r_{boundary}(x,t)}\left( \int _0^{2\pi } \, rd\phi \right) dr = {{\left( r_{boundary}(x,t) \right) ^2}\over {r_0^2}} = 1 - {{4 x \eta }\over {t\left( {{\Delta p}\over {L}}\right) r_0^2}} \end{aligned}$$

An interesting feature of this surprisingly simple result is that, within the region of the Poiseuille profile, there is a linear relationship between *x* and $f_R(x,t)$.
